# Unmet dental care needs and reported reasons: a comparison between adults with and without disabilities

**DOI:** 10.3389/froh.2025.1597916

**Published:** 2025-10-02

**Authors:** Huabin Luo, Melissa Stancil, Mark E. Moss

**Affiliations:** 1 Department of Public Health, Brody School of Medicine, East Carolina University, Greenville, NC, United States; 2 Department of Public Health, College of Pharmacy & Health Sciences, Campbell University, Buies Creek, NC, United States; 3 Department of Foundational Sciences, School of Dental Medicine, East Carolina University, Greenville, NC, United States

**Keywords:** unmet dental needs, disability, disparity, reasons of unmet dental care needs, dental utilization

## Abstract

**Objectives:**

To assess disparities in unmet dental care needs between adults with and without disabilities and the reasons for not being able to get needed dental care.

**Methods:**

We analyzed data from the 2015–2018 National Health and Nutrition Examination Surveys (NHANES). The main outcome variable was unmet dental care needs (yes/no)—not being able to get dental care when needed in the past 12 months. The independent variable, disability status, was classified by whether an individual has serious difficulty in conducting any of the following six activities: hearing, seeing, mobility, self-care, cognition, or independent living. The analytical sample included 11,288 adults ages 20 years and older. We used multiple logistic regression to assess the association between disability status (measured by any disability [yes/no], six different types[yes/no], and number of disabilities) and unmet dental care needs. The differences in reasons for not being able to get needed dental care were also assessed by disability status. Data analysis accounted for the complex survey design of NHANES.

**Results:**

Adults with any disability were more likely to have experienced unmet dental needs (AOR = 1.99, 95% CI: 1.71–2.33) than those without disabilities. There was a linear relationship between the number of disabilities and higher odds of experiencing unmet dental care needs (*P* < .01). The top two reasons for not being able to get needed dental care were “Cannot afford the cost” and “Insurance did not cover.”

**Conclusions:**

We found adults with disabilities had experienced more unmet dental needs. Further assistance including providing dental insurance coverage and mobile dental clinics is needed to help this disadvantaged population in access to dental care.

## 1 Introduction

Disability refers to any condition of physical and/or mental impairment that makes it more difficult for the person with the condition to do certain activities and interact with the world around them (1). More than 65 million or 27.2% of adults in the United States live with disabilities (2). Oral health is fundamental for overall health and well-being (3). Yet dental care is one of the most cited unmet needs for individuals with disabilities (4, 5). Individuals with disabilities have more limited access to dental visits than those without disabilities (6–10), which accounts for the poor oral health status in this population (5, 11–13).

Although a few studies examined self-reported unmet dental care needs among persons with disabilities (6–10), little research has assessed whether disparities in unmet dental care needs varied by different types of disabilities (mobility disabilities vs. vision disabilities). And reasons for unmet dental care needs in this population are not well-known. A better knowledge of specific reasons for unmet dental care needs for people with disabilities can inform efforts to address the barriers that limit access to dental care in this vulnerable population. Such data are essential for eliminating oral health disparities and achieving oral health equity.

This study aimed to address the aforementioned literature gaps. The study objectives were to assess the disparities in unmet dental care needs between adults with and without any disability, the associations of different types of disabilities and number of disabilities with unmet dental care needs, and the differences in reasons for not being able to get needed dental care.

## 2 Materials and methods

### 2.1 Data

Data were from the 2015–2018 National Health and Nutrition Examination Surveys (NHANES). The NHANES is a stratified multistage probability sample of the civilian non-institutionalized population in the United States. The NHANES includes both interviews and physical examinations. The interviews include socio-demographics, disability, and health service utilization, including dental care (14). The data used in this analysis were mainly from the Oral Health Module and the Disability Modules. The data are in the public domain and IRB review is exempted. The final analytical sample included 11,288 adults (ages ≥20 years, 5,719 in 2015–2016 NHANES, and 5,569 in 2017–2018 NHANES).

### 2.2 Measurement

#### 2.2.1 Outcome variables

1. Unmet dental care needs (Yes/No). Respondents were asked, “During the past 12 month, was there a time when you needed dental care but could not get it at that time?” Respondents were classified as having an unmet dental care need if they answered “Yes” to this question. Otherwise, they were classified as not having an unmet dental care need.
2. Reasons for unmet dental care needs. Respondents who answered they had experienced not being able to get the needed dental care in the past 12 months (the “unmet dental care needs” introduced above) were asked, “What were the reasons that you could not get the dental care needed?” There are 11 possible reasons for not being able to get the needed dental care: (a) Could not afford the cost, (b) Did not want to spend the money, (c) Insurance did not cover procedures, (d) Dentist office too far away, (e) Dentist office not open at convenient time, (f) Another dentist recommended not doing it, (g) Afraid of or do not like dentists, (h) Unable to take time off work, (i) Too busy, (j) Expected problems to go away, and (k) Other reasons. Participants can choose all reasons that apply.

#### 2.2.2 Independent variables

In the NHANES Disability Module, participants were asked whether they had the following six disabilities: (1) Deaf or serious difficulty hearing (hearing disability), (2) Blind or serious difficulty seeing even when wearing glasses (vision disability), (3) Serious difficulty concentrating, remembering, or making decisions (cognition disability), (4) Serious difficulty walking or climbing stairs (mobility disability), (5) Difficulty dressing or bathing (self-care disability), and (6) Difficulty doing errands alone, such as visiting a doctor's office or shopping (independent living disability). In this analysis, we measured this variable in three ways: (1) Any disability (one or none). Respondents were classified as having any disability if they answered “yes” to at least one of these six questions, as not having disability if they answered “no” to all six questions. (2) Six types of disability [all dichotomous, yes or none (without any disability)]. Respondents were classified as having the disability if they answered “yes” to the six disability type questions above. We recoded this variable so that the reference group is those without disabilities. (3) Number of disabilities. The sum of the six different types of disabilities, ranging from 0–6 disabilities. We combined 5 and 6 disabilities into one category because of small number of observations, and coded as 0, 1, 2, 3, 4, and 5 disabilities for this variable.

#### 2.2.3 Covariates

Following prior research (6–7, 9–10), We selected sociodemographic variables as covariates: Age (age groups 20–44, 45–64, and ≥65 years), sex, race/ethnicity (non-Hispanic White, non-Hispanic Black, Hispanics, non-Hispanic Asian Americans, and other race/ethnicity), education level (less than high school, high school, and some college and above), and family income level [<130% federal poverty level (FPL), 131% to 349% of FPL, and ≥350% FPL], assessed on the basis of the Poverty Index Ratio (PIR), the ratio of total family income to the U.S. poverty level, and health insurance coverage (Yes/No).

### 2.3 Statistical analysis

First the descriptive statistics of participant characteristics were calculated by the outcome variable—unmet dental care needs. Next, we conducted multiple logistic regression analyses to assess the association of disability status (any disability), different types of disabilities, and total number of disabilities with unmet dental care needs. Third, we assessed differences in reasons for unmet dental care needs. All analyses were conducted in Stata version 14.0 (Stata Corp, LLC) and accounted for the complex survey design of NHANES. We set significance at *P* = .05.

## 3 Results

### 3.1 Descriptive results

The prevalence of self-reported unmet dental care needs was higher in adults with disabilities than those without, 28.0% (95% CI: 25.7–30.4%) vs.15.4% (95% CI:14.2–16.8%) (*P* < .001). Table 1 presents the characteristics of respondents by unmet dental care needs. The prevalence of unmet dental care needs was higher in younger adults ages 20–44 years (21.9%), non-Hispanic Blacks (27.1%), those unmarried (23.1%), with less than high school education level (32.4%), with less than 130% FPL (36.0%), or without health insurance (41.7%) (all *P* < .001). It was also higher in females than in males (19.7% vs. 17.4%, *P* = .02).

**Table 1:** Characteristics of respondents by unmet dental care needs: NHANES 2015−2018.

Variables	Sample		Unmet dental needs	
	*n*	%	%	*p*
Age groups				<.001
20–44	4,522	44.3	21.9	
45–64	3,888	35.4	19.0	
65+	2,878	20.3	10.9	
Sex				0.015
Male	5,449	48.1	17.4	
Female	5,839	51.9	19.7	
Race/ethnicity				<.001
non-Hispanic white	3,798	63.1	15.3	
non-Hispanic black	2,496	11.4	27.1	
Hispanic	3,015	15.5	26.3	
Asian Americans	1,501	5.9	11.4	
Others	478	4.1	27.6	
Married				<.001
No	4,586	37.1	23.1	
Yes	6,693	62.9	16.0	
Education level				<.001
<High school	2,481	12.9	32.4	
High school	2,561	24.0	21.6	
Some college	3,470	31.6	21.0	
College or above	2,758	31.5	8.5	
Family income level				<.001
<130% FPL	3,004	20.6	36.0	
131%−350% FPL	3,991	36.3	21.7	
>=350 FPL	2,865	43.1	7.4	
Having health insurance				<.001
No	1,818	13.5	41.7	
Yes	9,442	86.5	15.1	

%, weighted percentage.

### 3.2 Logistic regression model results

Table 2 presents the logistic regression model results. In Model I, only adjusting for demographic variables, adults with any disability were more likely to have experienced unmet dental care needs (AOR [adjusted odds ratio] = 2.61, 95% CI: 2.27–3.01). In Model II, further adjusting for SES variables, the odds ratio for any disability decreased, but remained statistically significant—AOR = 1.99, 95% CI: 1.71–2.33. Overall, older adults ages 65 years or older (AOR = 0.40, 95% CI: 0.32–0.50) were less likely to report unmet dental care needs than those ages 20–44 years. Females were more likely to report unmet dental care needs (AOR = 1.18, 95% CI: 1.05–1.33) than males. Non-Hispanic Blacks (AOR = 1.29, 95% CI: 1.12–1.47) were more likely, while Asians (AOR = 0.70, 95% CI: 0.55–0.88) were less likely to have reported that they had unmet dental care needs in comparison with non-Hispanic whites. Adults with more education (i.e., high school education or above), higher family income or health insurance coverage were less likely to have experienced unmet dental needs (all *P* < 0.001).

**Table 2:** Logistic regression model results of factors associated with unmet dental needs: NHANES 2015–2018.

Variables	Model I				Model II			
	AOR	95% CI		*p*	AOR	95% CI		*p*
Any disability	2.61	2.27	3.01	<.001	1.99	1.71	2.33	<.001
Age groups (vs. 20–44)								
45–64	0.81	0.66	0.98	0.03	0.92	0.73	1.15	0.44
65+	0.33	0.28	0.39	<.001	0.40	0.32	0.50	<.001
Female	1.16	1.01	1.32	0.03	1.18	1.05	1.33	0.01
Race/ethnicity (vs. non-Hispanic White)								
non-Hispanic Black	1.83	1.58	2.13	<.001	1.29	1.12	1.47	<.001
Hispanic	1.78	1.51	2.09	<.001	0.97	0.80	1.18	0.79
Asian Americans	0.74	0.58	0.95	0.02	0.70	0.55	0.88	<.001
Others	1.75	1.27	2.42	<.001	1.27	0.85	1.89	0.23
Married	0.74	0.64	0.85	<.001	0.89	0.77	1.03	0.11
Education level (vs. <HS)								
High school					0.70	0.57	0.87	<.001
Some college					0.83	0.69	0.99	0.04
College or above					0.48	0.38	0.61	<.001
Family income level								
(vs. < 130% FPL)								
131%–350% FPL					0.69	0.60	0.80	<.001
>=350 FPL					0.28	0.21	0.37	<.001
Having health insurance					0.41	0.34	0.51	<.001

AOR, adjusted odds ratio; HS, high school; FPL, federal poverty level.

We conducted additional analyses by family income level and by insurance status by running same models introduced above. By family income level, the model results show that the AOR for any disability decreased from 2.28 (*p* < .001) among the sample of participants with a family income at FPL < 130% (the lowest level) to 1.84 (*p* < .001) among the sample with a family income at FPL131%–349%, to 1.54 (*p* < .09) among the sample with a family income at FPL > 350% (the highest level). By insurance status, the model results show that the AOR for any disability decreased from 2.32 (*p* < .001) in the sample without health insurance to 1.92 (*p* < .001) in the sample with health insurance (*P* < .001) (Data not shown in Table 2). Thus, these results indicate that a higher family income and health insurance can alleviate the risk of unmet dental care needs among individuals with any disability.

Table 3 presents logistic regression model results by the six different types of disability (i.e., six separate models) and the total number of disabilities. All the six different types of disabilities were associated with higher odds of experiencing unmet dental care needs, the AORs range from 1.95 (for hearing disabilities) to 2.74 (for self-care disabilities). In addition, there is a linear relationship between the number of disabilities and experiencing unmet dental care needs, AOR increased from 1.55 for one disability to 4.17 for five or more disabilities (All *P* < .001).

**Table 3:** Logistic regression model results of unmet dental care needs by types and number of disabilities: NHANES 2015–2018.

Independent variables in different Models	AOR	95% CI		*p*
Hearing disability	1.95	1.52	2.50	<.001
Vision disability	2.43	1.88	3.13	<.001
Cognitive disability	2.69	2.32	3.11	<.001
Mobility disability	2.35	1.87	2.95	<.001
Self-care disability	2.74	1.90	3.96	<.001
Independent living disability	2.19	1.82	2.64	<.001
Number of disabilities				<.001
1	1.55	1.23	1.94	<.001
2	2.34	1.83	3.00	<.001
3	2.58	1.79	3.74	<.001
4	3.03	1.91	4.80	<.001
5	4.17	2.27	7.67	<.001

The reference group is those without disabilities in all the models.

All models included covariates age, sex, race, marital status, education, income, and health insurance.

### 3.3 Reasons for unmet dental care

Table 4 presents the reasons for unmet dental care. Overall, the rankings of reasons for unmet dental care by disability status were not statistically significant (Wilcoxon test *P* = 0.66). The top two reasons for both groups were “Cannot afford the cost” (Rank #1), answered by 78% of adults with any disability and 75% of adults without any disability, and “Insurance did not cover” (Rank #2), answered by 28% and 29%, respectively. The ranking positions of reasons from #3 to #10 varied between the two groups. For example, “Other reasons” was ranked #3 in those with disabilities (10.4%), while it was ranked #5 in those without disabilities (6.8%).

**Table 4:** Frequency of reasons for not being able to get dental care by disability status: NHANES 2015–2018.

Reasons	Adults with disability					Adults without disability					*p*
	*n*	%	95% CI		Rank	*n*	%	95% CI		Rank	
Could not afford the cost	739	74.5	71.1	77.7	1	1,019	71.1	66.9	75.0	1	0.112
Insurance did not cover	307	27.5	23.4	32.1	2	343	24.5	20.2	29.4	2	0.282
Other reasons	125	10.4	8.2	13.0	3	123	8.9	6.8	11.7	5	0.433
Afraid or do not like dentists	80	10.0	7.1	14.0	4	86	7.2	5.1	10.2	7	0.090
Don't want to spend the money	56	6.8	4.9	9.4	5	120	11.2	8.6	14.3	3	0.003
Expected problems to go away	53	5.8	4.2	8.0	6	100	6.9	5.4	8.8	8	0.440
Too busy	54	5.3	3.7	7.5	7	111	7.8	5.8	10.5	6	0.089
Office not open at convenient time	41	4.5	3.0	6.6	8	45	2.9	2.1	4.0	9	0.065
Dental office too far away	43	4.2	2.6	6.8	9	45	2.9	2.0	4.2	10	0.238
Unable to take time off	34	3.0	1.9	4.8	10	122	9.7	7.6	12.3	4	0.000
Another dentist recommended not doing it	8	0.5^a^	0.2	1.2	11	11	0.5^a^	0.3	0.9	11	0.898

a Estimate not valid because the small sample size resulted in relative standard errors of more than 30%.

In addition, Chi-square tests of specific reasons showed statistically significant differences in the rankings for 2 out of the 11 reasons: “Do not want to spend the money”: 6.8% in adults with disabilities vs. 11.2% in those without (*P* = .003); and “Unable to take time off”, 3.0% in adults with disabilities vs. 9.7% in those without disabilities (*P* < .001). Finally, the reason “Afraid or do not like dentists” was ranked #4 in adults with disabilities while ranked #7 in adults without, 10.0% vs. 7.2% (*P* = 0.09).

## 4 Discussion

This study aimed to examine disparities in unmet dental needs between adults with and without disabilities and identify the most common reasons for unmet dental care needs in adults with disabilities. Analyzing the 2015–2018 NHANES data, we found that adults with disabilities were more than twice as likely to have experienced unmet dental care needs in comparison with those without. If it is a prolonged delay or they have to forgo dental treatment, it can lead to adverse outcomes given that a growing body of evidence indicates poor oral health may contribute to other diseases, such as diabetes (15), cardiovascular disease (16), and dementia (17–19). It is likely that adults with disabilities may delay dental care until it is urgently required. They may go to a hospital emergency room for treatment, which is costly and largely ineffective since definitive treatment requires care in a dental care setting. Further, they may have other competing healthcare priorities, may be unaware of need for dental care, and/or unwilling to visit a dentist. All these make them extremely vulnerable to oral health issues. Oral health education and oral care should be further promoted in adults with disabilities and their caregivers.

Although not directly comparable due to different measurement of disability status, our findings are in alignment with prior findings from other national surveys. For instance, an analysis (10) of the Medical Expenditure Pane Survey data showed that adults with intellectual and other disabilities were about 2.70 times the odds of being unable to get dental care compared with adults without disabilities.

By different types of disability, we found that the odds ratio of having unmet dental needs were all greater than 2 except for that of hearing disability. These findings suggest that mobility/transportation issue (i.e., self-care disability with the largest odds ratio, mobility disability with the third largest odds ratio) was the biggest barrier to access dental care. It may also suggest that dental clinics were not wheelchair accessible. In addition, there was a linear relationship between the number of disabilities and the likelihood of experiencing unmet dental care needs—the odds increased from 1.55–4.17. This suggests that people with multiple domains of disability experience greater challenges in obtaining needed dental care. The combination of sensory and mobility challenges among adults appears to raise significant barriers in seeking dental care. Community supports and mobile dental clinics that bring a well-trained oral health care team to places where people with disabilities live may reduce the dental care barriers for these individuals.

In general, we found that non-Hispanic Blacks were more likely to have unmet dental care needs than non-Hispanic whites. Adults with higher SES (income and education levels) were less likely to have unmet dental care needs. Individuals with disabilities from a lower income family had an even higher risk of unmet dental care needs, compared to their counterparts from a higher income family. Furthermore, we found that health insurance coverage may lower the risk of unmet dental care needs. Our findings are consistent with prior findings (20–23). It is interesting that adults ages 65 years and older were less likely to report having experienced unmet dental care needs compared to those ages 20–44 years. Another interesting finding is that Asian Americans were less likely to have unmet dental care needs than non-Hispanic whites. They tend to evaluate their oral health as better than it is. It should also be noted that even though disability status is a significant risk factor for unmet dental care needs, the SES variables appeared to have mitigated the impact of disability status. Thus, targeting SES can help to address disparities in oral health in adults and this would benefit those with disabilities as well as those without disabilities.

*Reasons for unmet dental care.* Overall, the predominant reasons for unmet dental care were financial barriers—the top two reasons were “Could not afford the cost” or “Insurance did not cover it” answered by 95% adults, regardless of disability status. Future policies should continue to address cost and coverage barriers to dental care among adults, particularly for low-income adults. Yet, in most states, Medicaid stops covering dental benefits for those above the age of 21 (24). This leaves many adults with disabilities with no access to affordable dental care. To eliminate dental care disparities for adults with disabilities, it is essential that Medicaid programs provide dental coverage for this population group. Concomitantly, it is essential that dental professionals are adequately reimbursed when caring for adults with disabilities in Medicaid programs.

Although slight difference was found between individuals with and without disabilities in the rankings of self-reported reasons, some results need further discussion. First, the third most common reason for those with disabilities was “Other reasons”, ranked #3, while it was ranked #5 in adults without disabilities. This catch-all category may hide important barriers specifically relevant to this population with disabilities. For instance, there might be provider-related barriers—Dental appointments being turned down due to the patient's disability status; lack of respect for patients with disabilities; (25) dentists being incompetent to manage adults with disabilities (4). A qualitative study is needed to explore and understand better the reasons behind unmet dental needs for people with disabilities, as well as identify barriers and facilitators. Second, “Afraid or do not like dentists” ranked #4 in adults with disabilities while those without disabilities ranked #7. This may suggest a need for case management and care coordination for people with disabilities since those with disabilities need more assistance to assuage their anxiety levels related to fear of dental treatment. Third, it is interesting that a significant higher proportion of adults without disabilities report they “Do not want to spend the money” compared to adults without disabilities. We do not know the reason behind these results.

## 5 Limitations

First, the data used for our study are cross-sectional. Thus, causality between disability and dental service utilization cannot be inferred. Second, disability estimates are likely underestimates because NHANES is only administered to non-insti­tutionalized adults and excludes institutionalized persons (e.g., nursing home residents), who might have higher disability prevalence and face more barriers to dental care. Thus, our estimates of unmet dental needs could be underestimated, and the reasons for unmet dental needs may be different. Third, data were self-reported and subject to recall and social desirability bias.

## 6 Conclusions

We found that adults with disabilities were more likely to have experienced unmet dental care needs than those without. Financial barriers are the top reasons for unmet dental care. Further assistance, including providing dental insurance coverage (e.g., expanding Medicaid dental coverage for adults with disabilities) and delivering dental care in the community (e.g., incentivizing mobile clinics or dental home models) is needed to help this vulnerable population access dental care.

## Data Availability

The data used in the are in the public domain. It is available at https://wwwn.cdc.gov/nchs/nhanes/Default.aspx.
